# Thirty-Year Glycemic Trajectories From Young Adulthood Through Middle Age

**DOI:** 10.1001/jamanetworkopen.2025.17455

**Published:** 2025-06-26

**Authors:** Abigail R. O. Arons, Lucia Pacca, David R. Jacobs, Anusha Vable, Dean Schillinger

**Affiliations:** 1 Division of General Internal Medicine at Zuckerberg San Francisco General, Department of Medicine, University of California, San Francisco; 2 Division of General Pediatrics, Department of Pediatrics, University of California, San Francisco; 3 Department of Family and Community Medicine, University of California, San Francisco; 4 Division of Epidemiology & Community Health, School of Public Health, University of Minnesota, Minneapolis

## Abstract

**Importance:**

Type 2 diabetes prevalence is increasing, and currently 24% of young adults have prediabetes. Little is known regarding timing and progression from normoglycemia to diabetes starting in young adulthood.

**Objective:**

To examine glycemic trajectories from young adulthood through middle age.

**Design, Setting, and Participants:**

This cohort study used sequence analysis to identify patterns of glycemic trajectories across 30 years in the Coronary Artery Risk Development in Young Adults (CARDIA) study, a large, diverse, population-based cohort of young adults from 4 national community sites with repeated measures of fasting plasma glucose (FPG). Sequence analysis reduces the various glycemic trajectories that individuals may follow over time into common trajectory clusters. The CARDIA study enrolled adults aged 18 to 30 years from 1985 to 1986, with groups balanced on Black and White race, sex, age, and educational level. Data through year 30 (2015-2016) were used, excluding participants with baseline diabetes, those missing the first 2 FPG values, and those missing 6 or more FPG values. Data analysis was performed from July 2023 to October 2024.

**Main Outcomes and Measures:**

Clusters of common glycemic trajectory patterns.

**Results:**

Among the 4684 study participants (2553 [54.5%] female), 1278 unique glycemic trajectories were identified across 30 years. A total of 3420 participants (73.0%) had trajectories classifiable into 9 common patterns of glycemic trajectory: stable normoglycemia; 5 patterns of impaired fasting glucose (IFG) not progressing to diabetes, including a brief period of low IFG regressing to normoglycemia at younger (mean [SD] age at IFG, 35.9 [6.6] years) and older ages (mean [SD] age at IFG, 46.8 [3.7] years), younger-onset sustained IFG (mean [SD] age at IFG, 38.6 [5.0] years), older-onset sustained low IFG (mean [SD] age at IFG, 51.7 [5.1] years), and older-onset sustained high IFG (mean [SD] age at IFG, 41.9 [8.8] years); and 3 patterns of diabetes, including young onset (mean [SD] age at diabetes, 34.8 [3.6] years), middle-age onset (mean [SD] age at diabetes, 43.6 [4.4] years), and older onset (mean [SD] age at diabetes, 52.4 [3.8] years).

**Conclusions and Relevance:**

This cohort study revealed substantial heterogeneity in glycemic trajectories across 30 years of follow-up. Clusters that represented IFG during young adulthood did not demonstrate progression to diabetes, and clusters that represented diabetes lacked prior IFG during young adulthood. These data suggest the need for strategies beyond simply screening for IFG during young adulthood to identify and target diabetes prevention for young adults.

## Key Points

**Question:**

How does the risk of diabetes progress from young adulthood through middle age in a diverse sample population?

**Findings:**

This cohort study of 5115 adults, beginning at 18 to 30 years of age and followed up for 30 years, revealed 9 predominant patterns of diabetes risk progression, including stable normoglycemia, 5 patterns of impaired fasting glucose that did not progress to diabetes, and 3 patterns of diabetes with onset at younger, middle, and older ages.

**Meaning:**

The results of this study call into question the utility of generalizing an impaired fasting glucose–based screening strategy informed by research in middle-aged populations to young adults and suggest a need for young adult–specific approaches to diabetes prevention.

## Teaser

This cohort study examines glycemic trajectories using sequence analysis in a population-based cohort of young adults with normoglycemia and prediabetes.

## Introduction

Young adulthood represents an unmet opportunity for diabetes prevention.^1,2^ A total of 24% of young adults have prediabetes,^3^ and diabetes prevalence has sharply increased across the lifespan, including aggressive early-onset diabetes.^4,5,6^ Robust evidence demonstrates that diabetes risk accumulates decades before diagnosis,^7^ and young adulthood is a unique developmental period of high plasticity.^8^ Underrepresentation of young adults in research hinders diabetes prevention efforts.^9,10,11^ With evidence lacking, the US Preventive Services Task Force has no diabetes screening guideline for adults younger than 35 years.^12,13^

Underpinning diabetes prevention efforts is an assumption that people progress from normoglycemia to prediabetes to diabetes. Guidelines recommend screening for prediabetes (with debate surrounding its precise definition) and offering preventive intervention, including intensive lifestyle modification and/or pharmacotherapy, to people with prediabetes.^12,13^ The actual path from normoglycemia to diabetes is more complex. People with prediabetes experience varying risks and timing of progression to diabetes, and some revert to normoglycemia.^14^ Age is one dimension of variation in the risk of progression: both adolescents^15,16,17^ and older adults^18,19^ with prediabetes are more likely to regress to normoglycemia than middle-aged adults. Young adulthood may also be distinct from other ages, given its unique physiology, psychology, and social circumstances.^8,20,21,22,23^

Glycemic trajectory analysis is useful to understand individuals’ paths to diabetes. Prior studies using middle-aged cohorts^24,25^ or universal adult cohorts without age stratification^26,27,28^ reveal heterogeneous patterns of progression of impaired fasting glucose (IFG) across short follow-up periods.^29^ Glycemic trajectory analysis has not been applied to young adults or across long periods. To better understand diabetes risk progression beginning in young adulthood, we characterized glycemic trajectories using sequence analysis in a large, diverse, population-based cohort of young adults with normoglycemia and prediabetes at baseline, using repeated measures of fasting plasma glucose (FPG) collected across 30 years.^30^

## Methods

### Study Population

The Coronary Artery Risk Development in Young Adults (CARDIA) study is an observational, longitudinal cohort study of 5115 young adults aged 18 to 30 years at baseline, with balanced subgroups by race (self-identified as Black, not Hispanic or White, not Hispanic, with those reporting any other race/ethnicity excluded), sex, age, and educational level. CARDIA’s sampling and measurement methods have been previously described in detail.^31^ CARDIA is notable among cohort studies for its frequent measurements of glycemic indicators beginning in young adulthood. The institutional review boards at each CARDIA study site approved the study procedures. All participants provided written informed consent. The study followed the Strengthening the Reporting of Observational Studies in Epidemiology (STROBE) reporting guideline.

We used data from baseline (1985-1986) through year 30 (2015-2016), representing 7 examinations (0, 7, 10, 15, 20, 25, and 30 years after baseline). Year 35 (2020-2022) was excluded from the main trajectory analysis due to a high rate of missing examinations but included in a bivariate analysis described later. We excluded people with diabetes at baseline (n = 35), those missing 6 or more FPG values (n = 375), and those missing FPG values for both examination years 0 and 7 (n = 18). We used FPG as the indicator of glycemic status because this measure was collected for more examination years than other glycemic indicators.

### Identifying Glycemic Trajectory Patterns

We characterized glycemic trajectory patterns over time using sequence analysis and cluster analysis.^30,32^ This method groups similar glycemic trajectories while maintaining substantive differences in order, duration, and timing of glycemic states among groups. First, we created individual-level glycemic trajectories. Second, we calculated a dissimilarity measure to quantify differences among individual trajectories using sequence analysis. Third, we used cluster analysis to group similar trajectories.

#### Creating Individual-Level Glycemic Trajectories

For each participant, we categorized FPG values into 7 mutually exclusive categories based on American Diabetes Association criteria^33^: normoglycemia (FPG <100 mg/dL [to convert to millimoles per liter, multiply by 0.0555]), low IFG (FPG of 100-109 mg/dL), high IFG (FPG of 110-125 mg/dL), diabetes (FPG >126 mg/dL or taking antidiabetic medication), pregnant, missing, and deceased. We subdivided low and high IFG because the World Health Organization uses the more restrictive criteria of high IFG only,^34^ with evidence suggesting the high IFG group experiences greater cardiovascular disease risk than the low IFG group.^14^ We performed sensitivity analysis using additional glycemic indicators (glycated hemoglobin and oral glucose tolerance test) when available.

We used exact antidiabetic medication names when available (years 0-15) and otherwise relied on self-report of diabetes medications (years 20, 25, and 30). Whether antidiabetic medication was used was asked only of those reporting a diabetes diagnosis, making it unlikely that participants were taking these medications for other indications. Participants with less than 480 minutes fasting at the time of plasma glucose (PG) measurement were categorized as normoglycemia (PG <100 mg/dL), missing (PG of 100-200 mg/dL), or diabetes (PG >200 mg/dL).^33^ Participants were categorized as pregnant for examinations during pregnancy and deceased for all scheduled examinations after their death dates.

Given the age balance in CARDIA with approximately equal numbers of participants ages 18 to 24 years and 25 to 30 years at baseline, to ensure trajectories would reflect glycemic status at comparable ages, we calibrated participants’ ages by shifting the older group (ages 25-30) to align with the subsequent examination in the younger group (eFigure 1 in Supplement 1). This resulted in a missing examination at either the beginning (for the older group) or end (for the younger group) of the sequence, which we imputed as described later. We conducted sensitivity analysis restricted to only the younger subgroup.

To impute missing data, we carried forward diabetes (but not normoglycemia or IFG, given our interest in progression to diabetes or reversion to normoglycemia among those with IFG) for subsequent missing examinations. We carried backward normoglycemia for initial missingness created by the age calibration. The remaining initial and internal missingness was categorized as missing, whereas terminal missingness was retained by permitting sequences of different length.

#### Sequence Analysis to Quantify Differences Among Individual Trajectories 

After constructing individual trajectories, we quantified differences among trajectories by comparing every trajectory with all other trajectories in the dataset. Differences were measured by assigning a penalty to each insertion, deletion, or substitution required to make 2 trajectories equivalent (eFigure 2 in Supplement 1). To quantify the minimum penalty to transform each trajectory into all other trajectories, we used the transitions-based substitution matrix with the Optimal Matching algorithm,^30,35^ with sensitivity analysis using an alternative theory–based penalty. The outcome of sequence analysis is a square, symmetric distance matrix of each trajectory’s dissimilarity to every other trajectory, with smaller distances representing more similar trajectories.

#### Cluster Analysis to Group Similar Trajectories

We used agglomerative, hierarchical clustering with Ward linkage (which minimizes within-cluster homogeneity and maximizes between-cluster heterogeneity) to group similar trajectories based on the values in the distance matrix.^36^ We applied Duda-Hart cluster stopping rules to determine the optimal number of clusters. We selected the cluster solution that maximized the Je(2)/Je(1) index and minimized its T squared.^37^ We validated the cluster solution using average silhouette width.^38^

#### Descriptive Analysis of Trajectories

For each cluster, we characterized the glycemic sequence pattern. We also described sociodemographic characteristics associated with diabetes risk: sex, family history of diabetes, mean baseline body mass index, and structural factors (race and parental educational level).

### Bivariate Analysis of Diabetes Outcomes by Age at Time of First IFG Examination

We conducted a separate bivariate analysis comparing age at the first IFG examination with age at the first diabetes examination. This analysis permitted evaluation of diabetes outcomes for the whole study sample (including those in artifact clusters due to missingness in the main analysis). With missingness less problematic, we included the year 35 examination data, extending outcomes by 5 years. We cross-tabulated categorical variables for age at first IFG and diabetes examinations, respectively, and performed χ^2^ testing. Age at first IFG was categorized as IFG before 35 years of age, IFG after 35 years of age, and no IFG (or none before diabetes) based on the US Preventive Services Task Force screening guidelines.^12^ Diabetes was categorized as diabetes before 40 years of age (early onset based on definition in prior work^39^), ages 40 to 50 years, older than 50 years, or never diabetes.

### Statistical Analysis

All analyses were conducted in Stata software, version 18 (StataCorp). All data cleaning and analysis code were checked by an independent reviewer. A 2-sided *P* < .05 indicates statistical significance. Data analysis was performed from July 2023 to October 2024.

## Results

Baseline characteristics of the analytic sample of 4684 individuals (91.6% of the CARDIA study population) are given in Table 1. Of the 4684 participants, 2553 (54.5%) were female and 2131 (45.5%) were male. A total of 2360 (50.4%) were Black and 2324 (49.6%) were White.

**Table 1.** Baseline Characteristics of the Study Sample

Characteristic	No. (%) of participants (N = 4684)
Sex	
Female	2553 (54.5)
Male	2131 (45.5)
Race and ethnicity	
Black	2360 (50.4)
White	2324 (49.6)
Father’s educational level^a^	
Less than high school	781 (16.7)
High school	1377 (29.4)
College or more	1682 (35.9)
Mother’s educational level^b^	
Less than high school	659 (14.1)
High school	1875 (40.0)
College or more	1786 (38.1)
Family history of diabetes	
Parent with history of diabetes	642 (13.7)
Sibling with history of diabetes	121 (2.6)
Combined history of diabetes in parent and/or sibling	722 (15.4)
Baseline BMI, mean (SD)	24.5 (5.0)

Abbreviation: BMI, body mass index (calculated as weight in kilograms divided by height in meters squared).

^a^ Data missing for 844 participants.

^b^ Data missing for 364 participants.

### Glycemic Trajectory Patterns

**Figure 1.**
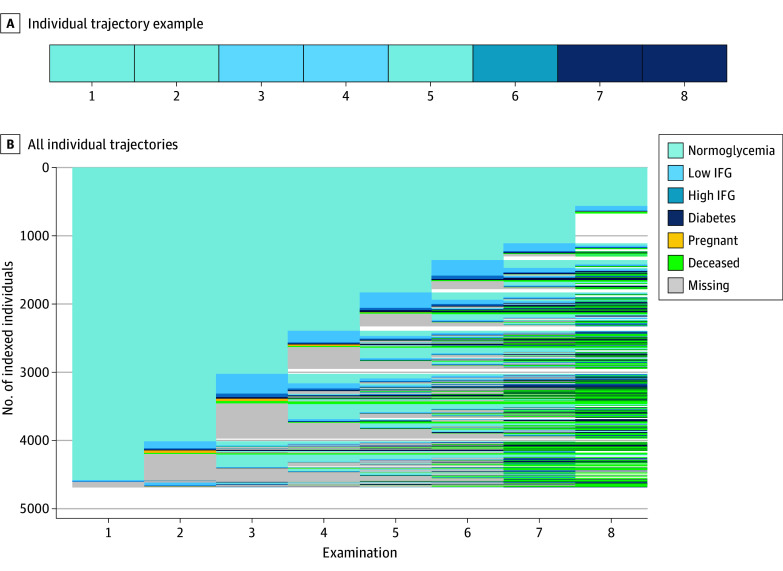
Plots of All Individual Trajectories for the 4684 Study Participants Individual-level glycemic trajectories represented in the Coronary Artery Risk Development in Young Adults (CARDIA) cohort from year 0 through year 30. A, An example trajectory of a single individual demonstrating fasting plasma glucose at each time point for examinations 1 through 8 categorized into 1 of 7 mutually exclusive glycemic states. B, Index plot compiling all 4684 observed glycemic trajectories (n = 1278 unique trajectories), with each individual’s trajectory as a row on the y-axis and each examination year represented on the x-axis. IFG indicates impaired fasting glucose.

We observed 1278 unique glycemic trajectories. The index plot of these trajectories revealed substantial heterogeneity in glycemic trajectories among individuals (Figure 1; eFigure 3 in Supplement 1). The sequence analysis resulted in 3420 participants (73.0% of the sample) being classified into 9 meaningful patterns of glycemic trajectory (described later). The remaining 1264 participants (27.0%) had unclassifiable glycemic trajectories, including individuals with long stretches of missing data before reemerging with diabetes (n = 81), those with early death (n = 69), and those with so many missing examinations that glycemic patterns were uninterpretable (n = 1114). Average silhouette width validated the cluster solution (eFigure 5 in Supplement 1).

#### Baseline Characteristics of Glycemic Trajectory Clusters

Baseline characteristics varied significantly among the 9 glycemic trajectory clusters (Table 2; eTable 1 in Supplement 1). Compared with the full sample, the normoglycemia cluster had a higher proportion of female and White participants. Among the IFG clusters not progressing to diabetes, the younger-onset brief low IFG cluster had the highest proportion of female participants, and the younger-onset sustained IFG cluster had the lowest proportion of female participants; all IFG clusters had more White than Black participants, except for the older sustained high IFG cluster. Both older sustained IFG clusters had a higher rate of family diabetes history than the other IFG clusters. Among the diabetes clusters, all had more Black than White participants. All diabetes clusters had lower rates of parental college education than the other clusters. The young diabetes cluster had higher rates of both sibling and parental diabetes than all other groups and the highest baseline body mass index of all clusters. All diabetes clusters had higher body mass index than the nondiabetes clusters.

**Table 2.** Characteristics of the Trajectory Clusters in the Classifiable Sample^a^

Characteristic	No. (%) of participants by cluster (N = 3420)^b^								
	Cluster 1: stable normoglycemia	Cluster 2: younger-onset brief low IFG	Cluster 3: older-onset brief low IFG	Cluster 4: younger-onset sustained IFG	Cluster 5: older-onset low IFG sustained	Cluster 6: older-onset high IFG sustained	Cluster 7: younger-onset diabetes	Cluster 8: middle-age–onset diabetes	Cluster 9: older-onset diabetes
Cluster size^c^	1619 (47.3)	391 (11.4)	142 (4.2)	194 (5.7)	265 (7.7)	380 (11.1)	72 (2.1)	180 (5.3)	177 (5.2)
Age at first IFG or diabetes examination, mean (SD), y^d^	NA	35.9 (6.6)	46.8 (3.7)	38.6 (5.0)	51.7 (5.1)	41.9 (8.8)	34.8 (3.6)	43.6 (4.4)	52.4 (3.8)
Sex^e^									
Female	1079 (66.6)	214 (54.7)	62 (43.6)	59 (30.4)	137 (51.6)	174 (45.7)	36 (50.0)	88 (48.8)	93 (52.5)
Male	540 (33.3)	177 (45.2)	80 (56.3)	135 (69.5)	128 (48.3)	206 (54.2)	36 (50.0)	92 (51.1)	84 (47.4)
Race and ethnicity^e^									
Black	701 (43.2)	176 (45.0)	59 (41.5)	73 (37.6)	95 (35.8)	190 (50.0)	52 (72.2)	121 (67.2)	118 (66.6)
White	918 (56.7)	215 (54.9)	83 (58.4)	121 (62.3)	170 (64.1)	190 (50.0)	20 (27.7)	59 (32.7)	59 (33.3)
Paternal educational level^e^									
Less than high school	262 (16.1)	58 (14.8)	17 (11.9)	34 (17.5)	33 (12.4)	67 (17.6)	11 (15.2)	49 (27.2)	39 (22)
High school	449 (27.7)	111 (28.3)	38 (26.7)	52 (26.8)	72 (27.1)	121 (31.8)	24 (33.3)	52 (28.8)	60 (33.8)
College or more	657 (40.5)	170 (43.4)	68 (47.8)	85 (43.8)	116 (43.7)	135 (35.5)	19 (26.3)	40 (22.2)	35 (19.7)
Maternal educational level^e^									
Less than high school	222 (13.7)	40 (10.2)	16 (11.2)	23 (11.8)	28 (10.5)	59 (15.5)	16 (22.2)	41 (22.7)	35 (19.7)
High school	628 (38.7)	140 (35.8)	49 (34.5)	81 (41.7)	97 (36.6)	159 (41.8)	25 (34.7)	78 (43.3)	74 (41.8)
College or more	674 (41.6)	185 (47.3)	67 (47.1)	80 (41.2)	114 (43)	136 (35.7)	24 (33.3)	44 (24.4)	47 (26.5)
Family history of diabetes^e^									
Parent history of diabetes	165 (10.1)	45 (11.5)	15 (10.5)	23 (11.8)	45 (16.9)	62 (16.3)	24 (33.3)	48 (26.6)	27 (15.2)
Sibling history of diabetes	32 (1.9)	7 (1.7)	2 (1.4)	7 (3.6)	13 (4.9)	4 (9.0)	9 (12.5)	8 (4.4)	3 (1.6)
Combined history of diabetes in parent and/or sibling	191 (11.7)	49 (12.5)	17 (11.9)	29 (14.9)	52 (19.6)	65 (17.1)	29 (40.2)	51 (28.3)	29 (16.3)
Baseline BMI, mean (SD)^e^	23.5 (4.4)	24 (4.4)	23.7 (3.8)	24.6 (4.5)	23.9 (4.1)	25.2 (4.8)	30.6 (5.5)	29.4 (6.5)	26.8 (5.6)

Abbreviations: BMI, body mass index (calculated as weight in kilograms divided by height in meters squared); CARDIA, Coronary Artery Risk Development in Young Adults; IFG, impaired fasting glucose; NA, not applicable.

^a^ Baseline (year 0) characteristics of the full sample and by trajectory cluster, demonstrating that those in IFG trajectories were largely similar to those in normoglycemic trajectories, whereas those in the diabetes trajectories were more socially vulnerable and had stronger family histories of diabetes. Of note, CARDIA’s sample population is balanced on race (Black and White), sex, and personal educational level at time of enrollment.

^b^ Cluster descriptions are as follows: (1) stable normoglycemia: normoglycemia throughout, (2) younger-onset brief low IFG: brief period of low IFG at younger ages, reverting to normoglycemia, (3) older-onset brief low IFG: brief period of low IFG at older ages, reverting to normoglycemia, (4) younger-onset sustained IFG: younger-onset low or high IFG that is sustained but did not progress to diabetes during the study period, (5) older-onset low IFG sustained: older onset of low IFG that is sustained but did not progress during the study period, (6) older-onset high IFG sustained: older onset of high IFG that is sustained but did not progress during the study period, (7) younger-onset diabetes: diabetes onset at younger ages, (8) middle-age–onset diabetes: diabetes onset at middle ages, and (9) older-onset diabetes: diabetes onset at older ages.

^c^ Percentage of total classifiable sample (N = 3420). All other percentages in the table are percentage of a given cluster.

^d^ Age in years at time of first CARDIA examination when IFG was detected (for clusters 2-6) or diabetes was detected (for clusters 7-9).

^e^ Difference across clusters is *P* < .001 by χ^2^ testing.

#### Glycemic Trajectory Patterns

**Figure 2.**
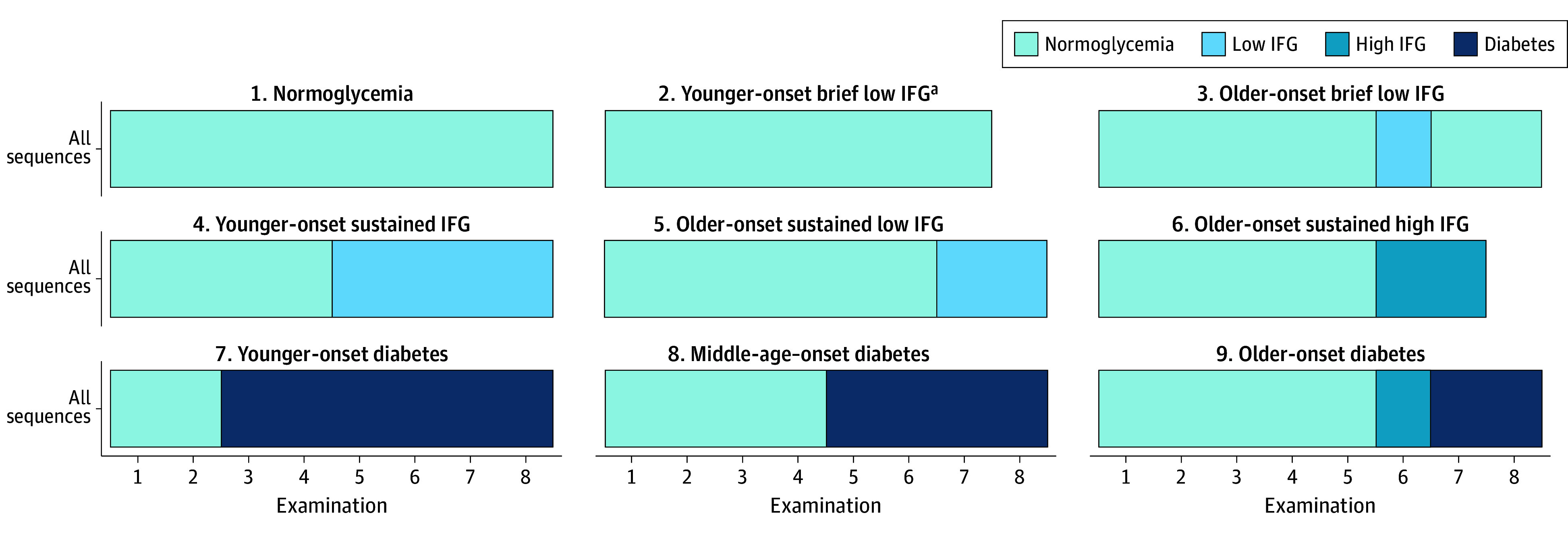
Modal Plot of the 9 Common Trajectory Patterns in the Classifiable Sample of 3420 Study Participants Modal plots of the 9 main glycemic trajectory patterns in the classifiable sample. Modal plots represent the most frequent state at each time point in the cluster. To define cluster labels and determine differences among visually similar modal plots, we examined additional visualizations of the clusters, including the chronogram and cluster-specific index plots (eFigure 4 in Supplement 1). ^a^In the younger-onset brief low impaired fasting glucose (IFG) cluster, a single examination of low IFG occurred at examination 1, 2, or 3 (see eFigure 4 in Supplement 1 for more details); because IFG was not the modal state at any one time point given the reversion to normoglycemia, the modal plot shows normoglycemia over the whole trajectory.

For the classifiable sample of 3420 individuals, the 9 patterns of glycemic trajectory (Figure 2; eFigure 4 in Supplement 1) included the following: stable normoglycemia; 5 patterns of IFG not progressing to diabetes, including a brief period of low IFG regressing to normoglycemia at younger (mean [SD] age at IFG, 35.9 [6.6] years) and older ages (mean [SD] age at IFG, 46.8 [3.7] years), younger-onset sustained IFG (mean [SD] age at IFG, 38.6 [5.0] years), older-onset sustained low IFG (mean [SD] age at IFG, 51.7 [5.1] years), and older-onset sustained high IFG (mean [SD] age at IFG, 41.9 [8.8] years); and 3 patterns of diabetes, including young onset (mean [SD] age at diabetes, 34.8 [3.6] years), middle-age onset (mean [SD] age at diabetes, 43.6 [4.4] years), and older onset (mean [SD] age at diabetes, 52.4 [3.8] years). Results were robust to sensitivity analyses restricted to the younger group, using alternative penalties and an expanded definition of diabetes (eFigures 6-8 in Supplement 1).

No trajectory had a modal pattern of people with IFG in young adulthood progressing to diabetes (they either reverted to normoglycemia or sustained IFG). Among the 3 diabetes trajectory patterns, only the older-onset diabetes trajectory aligned with the expected pattern of normoglycemia progressing to IFG progressing to diabetes; this group had normoglycemia during young adulthood, with IFG occurring later. The young and middle-age diabetes modal trajectories progressed directly from normoglycemia to diabetes without an interim measured IFG.

### Bivariate Analysis of Glycemic Categories Using Age

**Figure 3.**
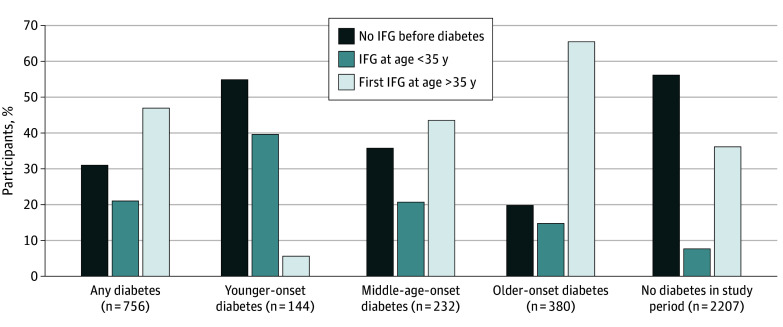
Results of Bivariate Analysis of Age at First Impaired Fasting Glucose (IFG) Examination by Age at First Diabetes Examination During 35 Years of Follow-Up *P* < .001 (χ^2^ test). Numerical results are given in eTable 2 in Supplement 1.

The bivariate analysis (Figure 3; eTable 2 in Supplement 1) confirmed the heterogeneity in diabetes outcomes seen in the trajectories, including those who were unclassifiable in the main analysis and extending the follow-up period by 5 years (to 35 years after baseline). Overall, 756 individuals had diabetes, with 519 (68.7%) having preceding IFG, most of which was first detected after 35 years of age (358 of 519 [69.0%]). Those with younger-onset diabetes (detected before 40 years of age) followed a markedly different pattern, more likely to lack preceding IFG (79 of 144 [54.9%]) than those with middle-age–onset diabetes (83 of 232 [35.8%]) and older-onset diabetes (75 of 380 [19.7%]) (*P* < .001). Less than half of those with younger-onset diabetes (57 of 144 [39.6%]) had IFG before 35 years of age. Of those not progressing to diabetes during the 35-year study period, 300 (7.6%) had IFG before 35 years of age, and 1421 (36.2%) had IFG at 35 years or older.

## Discussion

We sought to understand glycemic trajectories across 30 years starting in young adulthood in a large, diverse cohort. Overall, we found that glycemic trajectories based on FPG were widely heterogenous. We identified 9 common patterns of glycemic trajectory, including stable normoglycemia, 5 patterns of IFG not progressing to diabetes, and 3 patterns of diabetes with onset at younger, middle, and older ages. These trajectories are generally consistent with FPG trajectory studies (stable, regressing, and progressing) in middle-aged and universal cohorts.^25^ These results offer rare insight into short- and long-term diabetes risk beginning early in adulthood, given CARDIA's young adult cohort, the 30-year follow-up period, and our use of a novel visualization technique based on sequence analysis that, to our knowledge, has not been previously used to understand young adults’ glycemic trajectories. These new analyses can inform the approach to screening policy and targeted intervention to prevent diabetes.

Several notable findings emerged. First, clusters representing IFG during young adulthood either reverted to normoglycemia (younger-onset brief low IFG) or sustained IFG across the 30-year follow-up period, rather than predominant progression to diabetes, which was corroborated by the bivariate analysis. This finding is in contrast to the longest published follow-up study (control group with prediabetes at baseline had 30-year cumulative incidence of 96%^40^), although our finding is in line with other work with shorter follow-up.^41^ Second, the clusters progressing to diabetes lacked IFG during young adulthood. The younger-onset diabetes cluster progressed from normoglycemia directly to diabetes, corroborated by the bivariate analysis. This distinct pattern of risk is consistent with evidence that early-onset type 2 diabetes follows a rapidly progressive phenotype.^5,39^ Most individuals in the middle-age–onset and older-onset diabetes clusters had normoglycemia in young adulthood, again corroborated in the bivariate analysis. These findings point to 3 key questions: (1) How should those with IFG during young adulthood be risk stratified to target those at highest risk for progression given most do not progress to diabetes by middle age? (2) How can those at risk for younger-onset diabetes be identified given their lack of preceding IFG? (3) How can individuals at risk for middle-age–onset and older-onset diabetes during young adulthood be identified when most had normoglycemia?

Together, these insights and associated questions suggest that the course of diabetes risk during young adulthood is distinct from older ages and that relying on IFG during young adulthood to estimate future risk (ie, simply lowering the age for prediabetes screening to include younger adults) could miss many of those who will develop diabetes at both younger and older ages and overidentify others. With the advent of both pharmacologic^42^ and behavioral interventions^43^ that can modify trajectory, such misidentification risks reduced access to treatment for those at highest risk of progression, unnecessary intervention for those at lower risk, and imprecise estimation of treatment effects. In contrast, most of the older diabetes group followed the expected pattern of IFG preceding diabetes. That this pattern better aligns with the paradigm of prediabetes to diabetes progression is likely a reflection that the existing screening and prevention strategy is derived from studies conducted in middle-aged populations. Our results suggest that young adult diabetes prevention strategies cannot be generalized from these studies.

Instead, novel approaches to young adult diabetes prevention are warranted. Two broad considerations arising from our findings include risk stratification and population-based approaches. The baseline characteristic differences between clusters suggest that health and sociodemographic indicators could be incorporated alongside IFG to better approximate diabetes risk. For young adults not meeting blood-based prediabetes criteria, clinicians may consider using screening questionnaires^44^ to assess diabetes risk. Furthermore, novel biomarkers may improve early detection of diabetes risk in young adults over later-stage indicators of decompensating glucose regulation, such as IFG; future research should include validation of screening tools in this age, analyses of risk factor coevolution alongside glycemic trajectories, and dedicated prospective studies to elucidate young adult diabetes physiology (eg, metabolomics). Additionally, given the difficulty in identifying individual young adults at risk for diabetes, population-based approaches may be effective in targeting communities with high diabetes prevalence. Future research to understand neighborhood-level risk factors, with the essential inclusion of young adult perspectives throughout the research process, can inform such approaches.

### Limitations

Limitations of this study include that examinations occurred approximately every 5 years, so precise timing of IFG and diabetes onset could not be estimated and interval fluctuations in FPG between examinations were not reflected; these intervals may reflect actual screening frequency, especially in young adulthood. CARDIA began in 1985 to 1986, when population rates of IFG and diabetes were lower than today. Although some findings apply regardless of prevalence (eg, the rapid transition from normoglycemia to diabetes in early-onset diabetes), people with characteristics that 30 years ago would place them in lower-risk clusters today likely experience higher diabetes risk. CARDIA remains the most modern cohort study, to our knowledge, with frequent measures of glycemic status beginning in young adulthood; more recent cohort studies are needed. Our reliance on FPG may underestimate diabetes rates because FPG reflects a phenotype more tied to β-cell failure compared with glycated hemoglobin, which more closely tracks insulin resistance^45^ and has less intraindividual variability than FPG.^46^ Additional limitations include an upper mean age of 55 years at the last examination with likely later diabetes progression in some participants, potential for survivorship bias because many deceased participants were in artifact clusters, inclusion of only people of Black or White race (limiting generalizability), and inability to distinguish type 1 from type 2 diabetes, except by excluding people with diabetes at baseline. During the follow-up period, many individuals missed at least some examinations. Although we were able to impute some of this missingness, approximately one-quarter of individuals were missing, so many examinations fell into artifact trajectories, introducing selection bias because these individuals were more socially at risk. We likely underestimate risk of diabetes among those with higher social risk. These people were included in the bivariate analysis. Prior studies^24,25,26,27,28,29^ avoid these limitations with shorter, cumulative analyses; our findings add important longitudinal context.

## Conclusions

In this cohort study of a diverse population-based sample of young adults, we found substantial heterogeneity in glycemic trajectories across 30 years. Namely, clusters representing IFG during young adulthood did not demonstrate progression to diabetes, and clusters representing diabetes lacked IFG trailing back to young adulthood. Our results call into question the utility of generalizing the IFG-based screening strategy informed by research in middle-aged populations to young adults and reveal a need for young adult–specific approaches to diabetes prevention, including risk stratification tools and population-based interventions. The lack of a national diabetes screening and prevention strategy informed by and developed specifically for young adults presents an unmet opportunity for diabetes prevention.

## Notes

Supplement 1. **eFigure 1.** Age calibration of FPG exams between the younger and older groups at baseline enrollment **eFigure 2.** Assigning penalties in sequence analysis **eFigure 3.** Index plot for individuals with IFG at baseline **eFigure 4.** Full set of visualizations for the trajectory clusters **eFigure 5.** Average silhouette width plot **eFigure 6.** Sensitivity analysis with only younger group (18-24 at baseline with no age offset) **eFigure 7.** Sensitivity analysis using alternative penalty scheme (theory-based penalty) **eFigure 8.** Sensitivity analysis using expanded definition of diabetes (HgbA1c and 2hOGTT) **eTable 1.** Baseline characteristics of “unclassifiable” clusters **eTable 2.** Complete results of bivariate analysis

Supplement 2.
Data Sharing Statement
