# Supplementary material for: Thirty-Year Glycemic Trajectories From Young Adulthood Through Middle Age
Source: JAMA Netw Open. 2025 Jun 26;8(6):e2517455. doi: 10.1001/jamanetworkopen.2025.17455 (PMC12203282; doi:10.1001/jamanetworkopen.2025.17455)
Supplement: Supplement 1. — eFigure 1. Age calibration of FPG exams between the younger and older groups at baseline enrollment eFigure 2. Assigning penalties in sequence analysis eFigure 3. Index plot for individuals with IFG at baseline eFigure 4. Full set of visualizations for the trajectory clusters eFigure 5. Average silhouette width plot eFigure 6. Sensitivity analysis with only younger group (18-24 at baseline with no age offset) eFigure 7. Sensitivity analysis using alternative penalty scheme (theory-based penalty) eFigure 8. Sensitivity analysis using expanded definition of diabetes (HgbA1c and 2hOGTT) eTable 1. Baseline characteristics of “unclassifiable” clusters eTable 2. Complete results of bivariate analysis [file jamanetwopen-e2517455-s001.pdf]

Supplemental Online Content

Arons AR, Pacca L, Jacobs DR Jr, Vable A, Schillinger D. Thirty-year glycemic trajectories from young adulthood through middle age. *JAMA Netw Open*. 2025;8(6):e2517455. doi:10.1001/jamanetworkopen.2025.17455

eFigure 1. Age calibration of FPG exams between the younger and older groups at baseline enrollment

eFigure 2. Assigning penalties in sequence analysis

eFigure 3. Index plot for individuals with IFG at baseline

eFigure 4. Full set of visualizations for the trajectory clusters

eFigure 5. Average silhouette width plot

eFigure 6. Sensitivity analysis with only younger group (18-24 at baseline with no age offset)

eFigure 7. Sensitivity analysis using alternative penalty scheme (theory-based penalty)

eFigure 8. Sensitivity analysis using expanded definition of diabetes (HgbA1c and 2hOGTT)

eTable 1. Baseline characteristics of "unclassifiable" clusters

eTable 2. Complete results of bivariate analysis

This supplemental material has been provided by the authors to give readers additional information about their work.

© 2025 Arons AR et al. *JAMA Network Open.*

eFigure 1. Age calibration of FPG exams between the younger and older groups at baseline enrollment.

Mean Age (SD 2.0)	21.4	28.4	31.4	36.5	41.6	46.5	51.5	
**Baseline 18-24**								
Original Study Year	*Year 0*	*Year 7*	*Year 10*	*Year 15*	*Year 20*	*Year 25*	*Year 30*	***Age offset***
**Calibrated Exam #**	**1**	**2**	**3**	**4**	**5**	**6**	**7**	**8**
Mean Age (SD 1.8)		27.7	34.7	37.7	42.8	47.8	52.7	57.7
**Baseline 25-30**								
Original Study Year	***Age offset***	*Year 0*	*Year 7*	*Year 10*	*Year 15*	*Year 20*	*Year 25*	*Year 30*

This figure shows an example trajectory depicted if the individual were aged 18-24 at baseline, versus aged 25-30 at baseline. Mean ages for the two groups are shown, demonstrating comparable mean ages at a given calibrated Exam between the two groups under this offset. While splitting into just two groups meant the ages were not fully identical, each split into a more granular group for additional refinement of the age match-up would have generated an additional need for an offset exam and therefore additional missing values to be approximated; the two-group solution balanced both the need to standardize and the need to minimize missing exams.

Missing exams created by the age offsets were imputed as described in the text. After imputation, the age calibration created initial missingness for 71 of 2637 individuals with baseline age equal to or greater than 25 (2.7%). This included individuals who had a baseline exam other than normoglycemia (IFG or pregnant) or were already missing the baseline bloodwork. After imputation, age calibration created terminal missingness for 1708 of 2047 individuals with baseline age under 25 (83.4%), which included individuals without diabetes or death at the preceding exam. However this was less of a concern as the sequence method allowed for terminal missingness by permitting different lengths of sequences.

For all timepoints, in the Baseline 18-24 group, age standard deviation was between 1.99 and 2.04, and in the Baseline 25-30 group age standard deviation was between 1.74 to 1.81.

A sensitivity analysis with only the younger group is included in eFigure 6.

© 2025 Arons AR et al. *JAMA Network Open.*

eFigure 2. Assigning penalties in sequence analysis

	Person 1	Person 2
Time 1	A	A
Time 2	B	
Time 3	C	C

Penalty = 1 insertion →

	Person 1	Person 2
Time 1	A	A
Time 2	B	B
Time 3	C	C

	Person 1	Person 2
Time 1	A	A
Time 2	B	
Time 3	C	C

Penalty = 1 deletion →

	Person 1	Person 2
Time 1	A	A
Time 2		
Time 3	C	C

	Person 1	Person 2
Time 1	A	A
Time 2	B	A
Time 3	C	C

Penalty = 1 substitution →

	Person 1	Person 2
Time 1	A	A
Time 2	B	B
Time 3	C	C

In the second step of sequence analysis, each trajectory is compared to all other trajectories and a distance is calculated to quantify the differences between each pair of trajectories. Distances are the sum of penalties required to make one trajectory match another trajectory. This figure demonstrates the three possible ways a sequence would be modified to match another: insertion, deletion and substitution. In our analysis, the penalty for insertion or deletion is approximately half the maximum substitution penalty. The outcome of the sequence analysis step is a square, symmetric distance matrix of each trajectory's dissimilarity (minimum total penalty to match) to every other trajectory, with smaller distances representing more similar trajectories.

© 2025 Arons AR et al. *JAMA Network Open.*

eFigure 3. Index plot of subset of individuals with IFG at baseline (n=92)

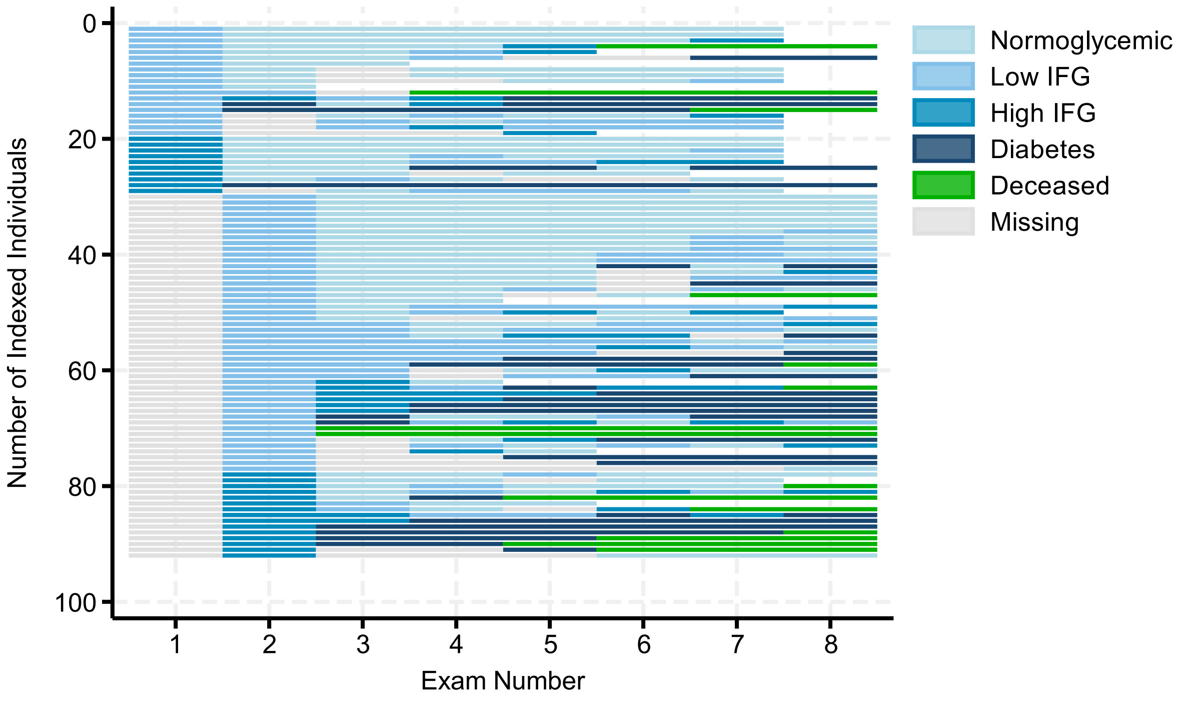

Index plot showing the glycemic trajectories of those individuals with Low or High IFG at their baseline exam (n=92). By design, Exam 1 is missing for those who were 25-30 years old at enrollment, as IFG was not pulled back to the preceding exam.

© 2025 Arons AR et al. *JAMA Network Open.*

eFigure 4. Full set of visualizations for the trajectory clusters

The sequence analysis algorithm produced 18 clusters which were manually consolidated into 9 trajectory patterns by combining three clusters of stable normoglycemia distinguished by missing a single exam into one “Stable Normoglycemia” pattern, and setting aside as “unclassifiable” one cluster of substantial missingness then diabetes, three “Deceased Early” clusters, three “Artifact” clusters. All 18 clusters are shown in the visualizations below.

eFigure4a. Modal plot of all trajectory clusters

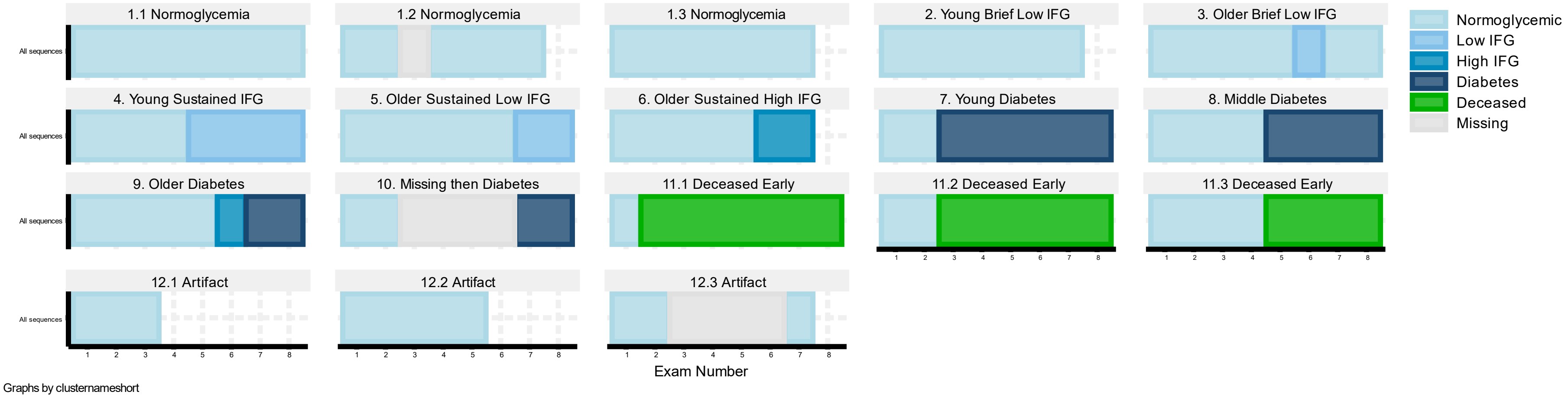

Modal plots of all 18 trajectory clusters. As described in the main text, Clusters 1.1, 1.2, and 1.3 were collapsed into a single Normoglycemia (Cluster 1) as they were distinguished only by if a single missing exam occurred (see index plots in eFigure 3c for additional clarification). For the purpose of aggregating baseline characteristics, Clusters 11.1, 11.2 and 11.3 were collapsed into a single Deceased Early cluster, and Clusters 12.1, 12.2 and 12.3 were collapsed into a single Artifact cluster.

© 2025 Arons AR et al. *JAMA Network Open.*

eFigure4b. Chronogram of all trajectory clusters

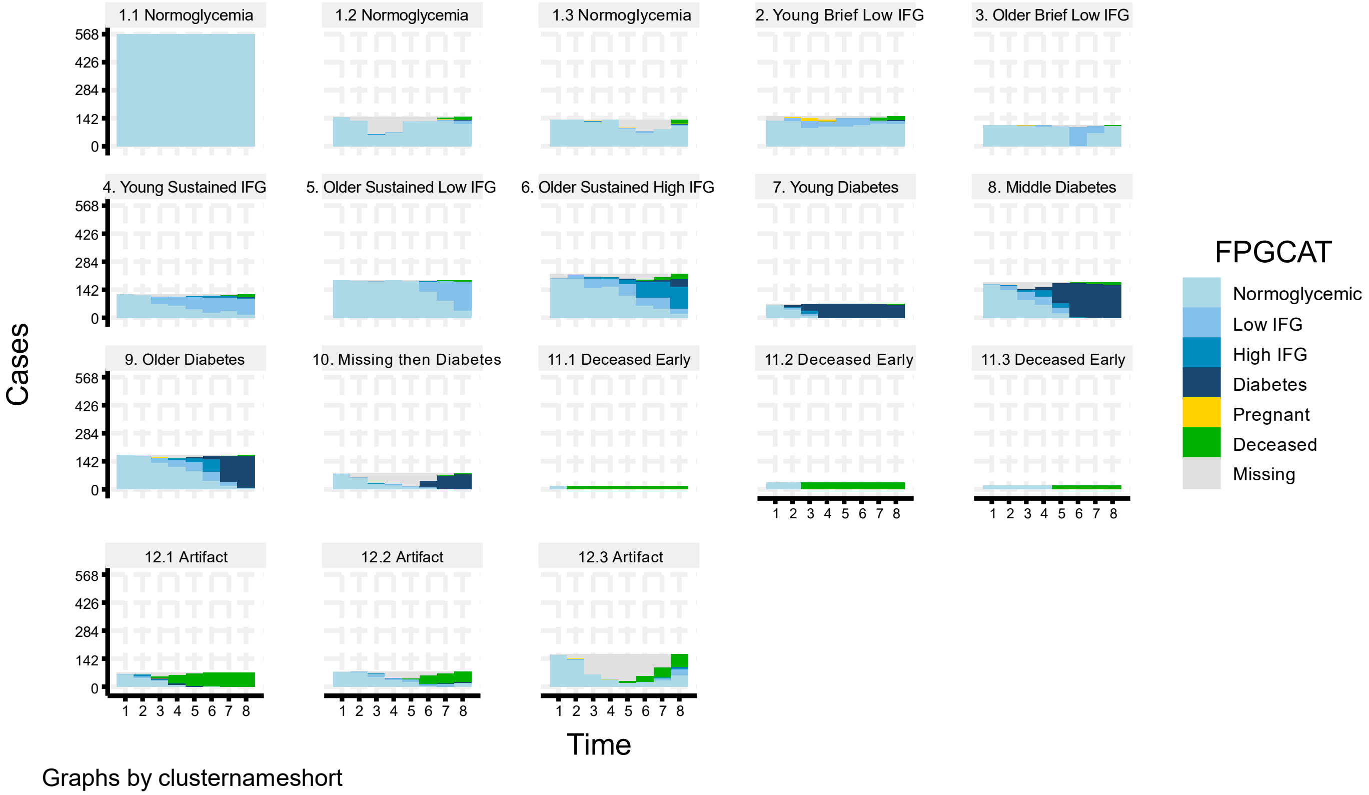

At each exam, this chronogram depicts a vertical bar showing the distribution of FPG categories at that exam. The y-axis is the number of individuals in that cluster, such that the relative heights of the clusters visually represents the relative proportion of the study population in that cluster.

© 2025 Arons AR et al. *JAMA Network Open.*

4c. Index Plots of All Clusters

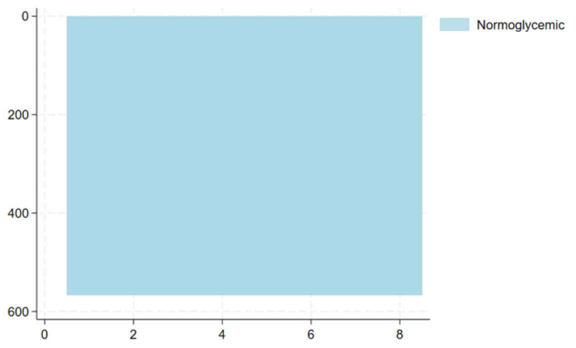

1.1 Normoglycemia

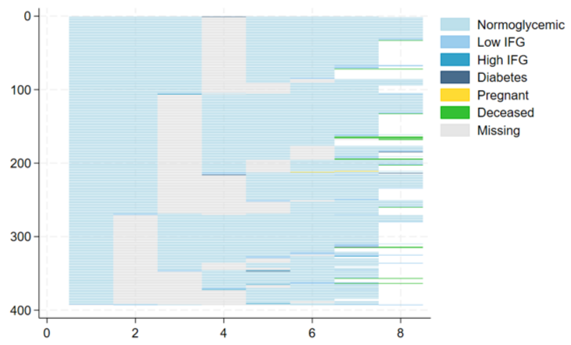

1.2 Normoglycemia

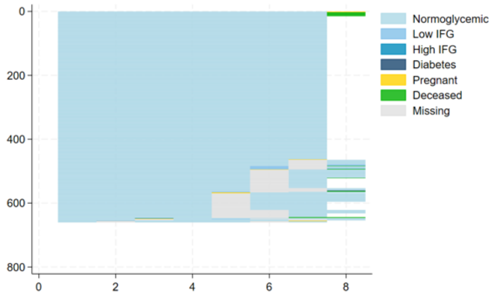

1.3 Normoglycemia

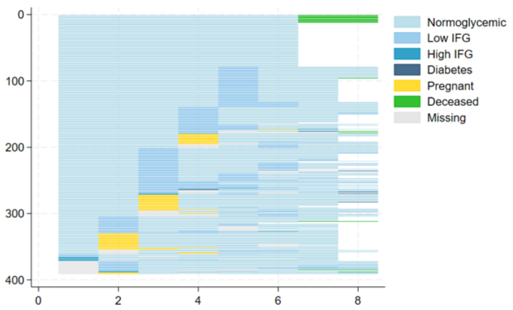

2. Young Brief Low IFG

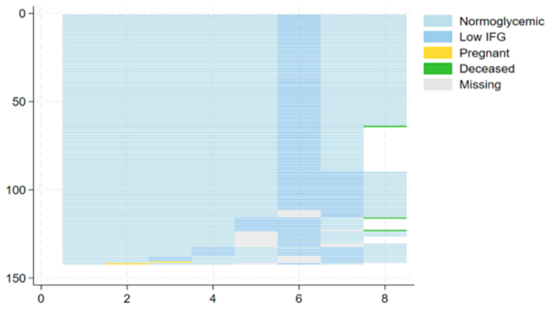

3. Older Brief Low IFG

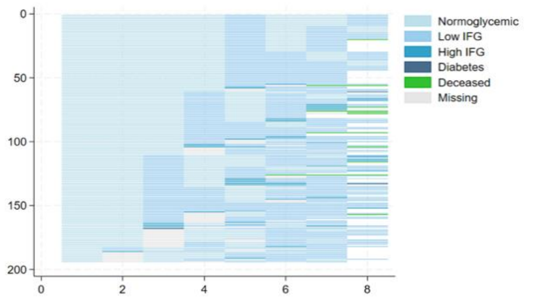

4. Young Sustained IFG

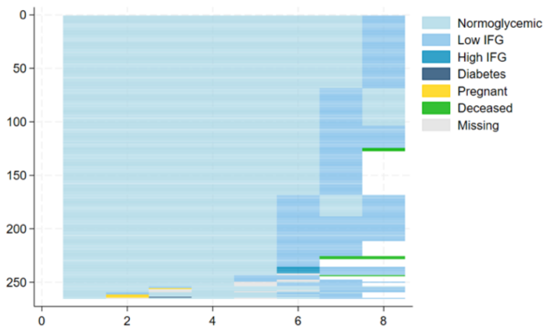

5. Older Sustained Low IFG

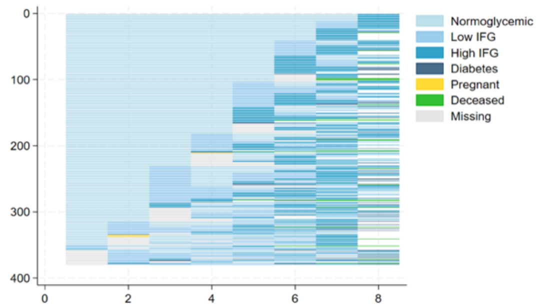

6. Older Sustained High IFG

© 2025 Arons AR et al. *J.*

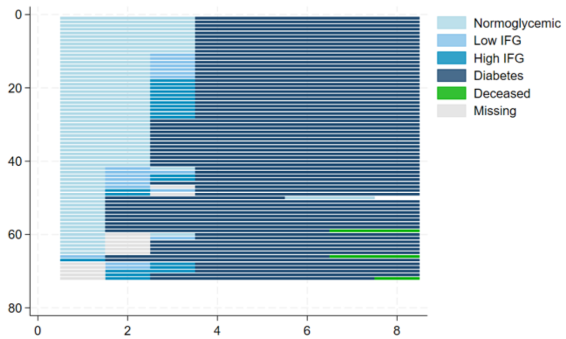

7. Young Diabetes

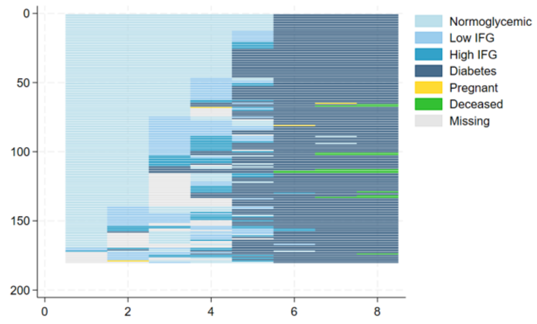

8. Middle Diabetes

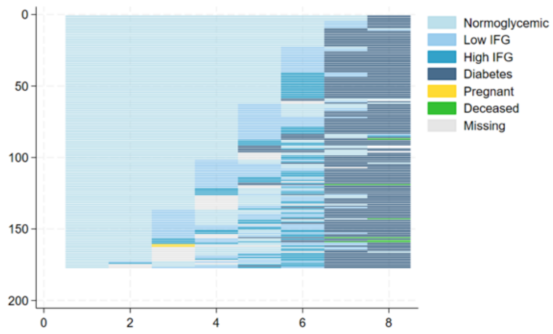

9. Older Diabetes

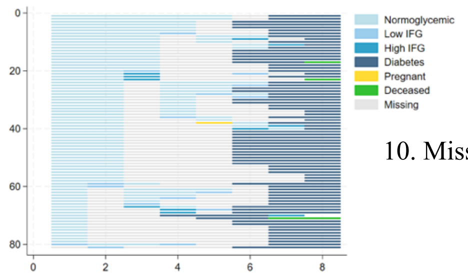

10. Missing then Diabetes

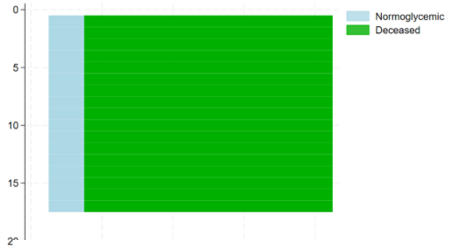

11.1. Deceased Early

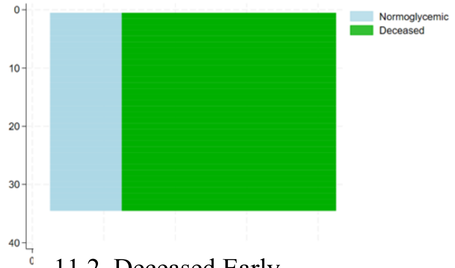

11.2. Deceased Early

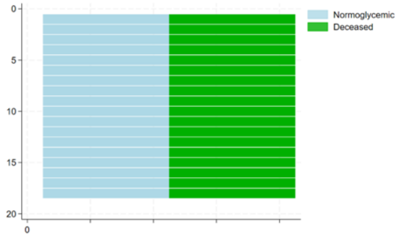

11.3. Deceased Early

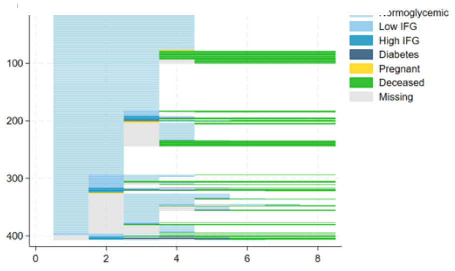

12.1. Artifact

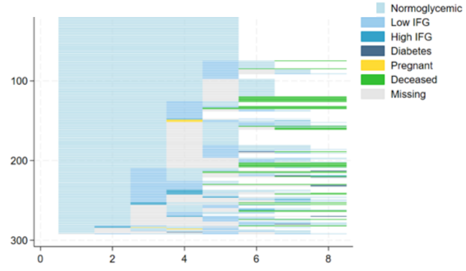

12.2. Artifact

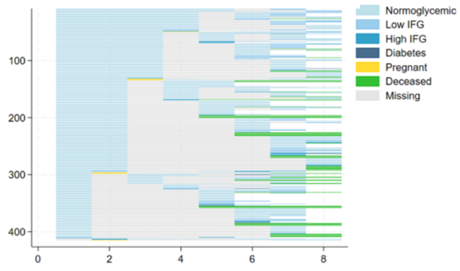

12.3. Artifact

© 2025 Arons [illegible] n.

eFigure 4d. Dendrogram

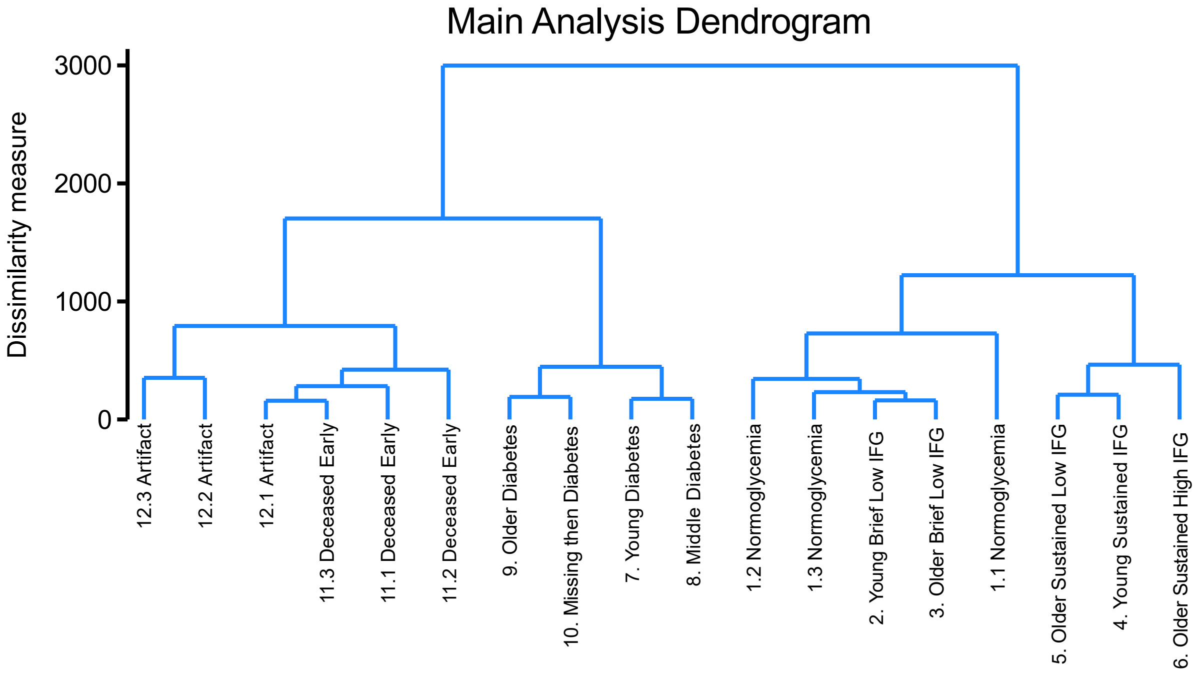

A dendrogram is a tree-like diagram that visually represents the results of hierarchical clustering, showing how individual trajectories are grouped based on their similarity from the distance matrix. Smaller height of the vertical lines connecting two clusters together indicate more similar clusters.

© 2025 Arons AR et al. *JAMA Network Open.*

eFigure 5. Average Silhouette Width

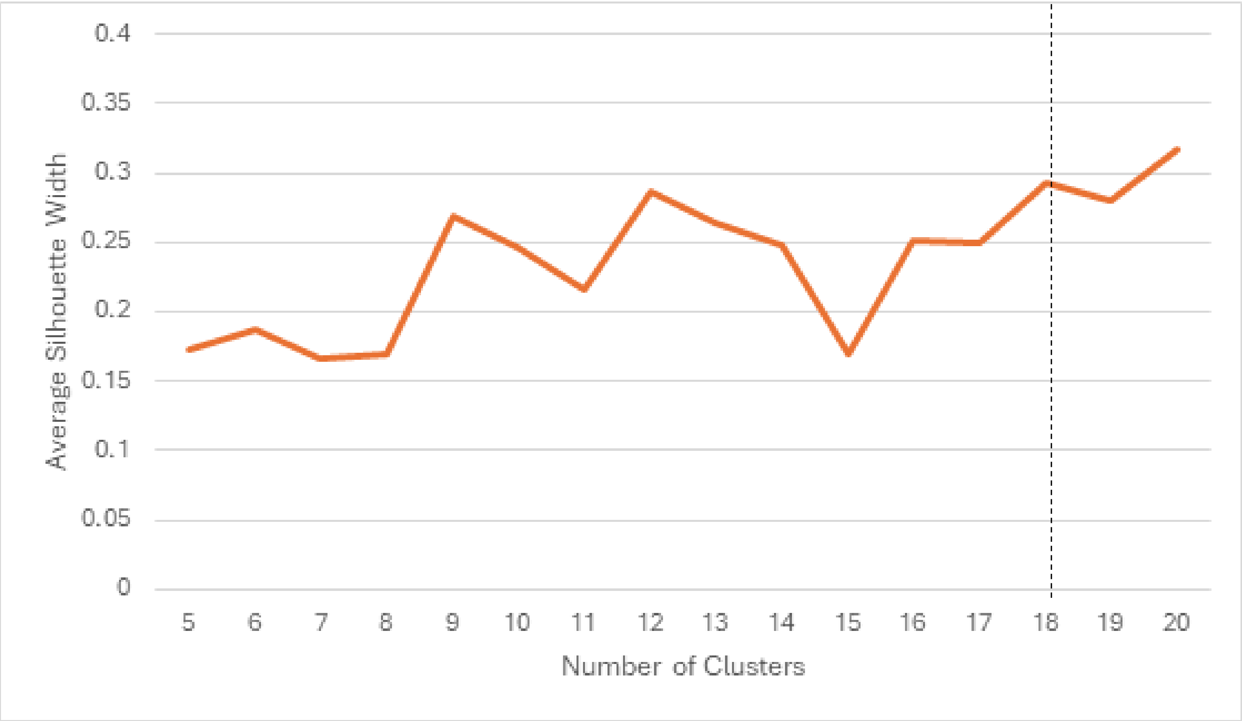

Plot of Average Silhouette Width metrics (ASW) for solutions from 5 to 20 clusters. ASW is a measure that compares the average distance of each observation within a cluster from other observations its own cluster, and to observations in the next nearest cluster. The maximum ASW indicates the optimal cluster solution, with a threshold of 0.25 considered sufficient for sequence analysis clusters. In this case, the maximum ASWs were for 20 clusters (ASW 0.317), 18 clusters (ASW 0.293) and 12 clusters (ASW 0.286), with all other cluster solutions having ASW of 0.27 or below. This validates the Duda Hart solution of 18 clusters as presented in the main analysis.

eFigure 6. Sensitivity analysis with only younger group (18-24 at baseline with no age offset n=2047)

A. Modal plot

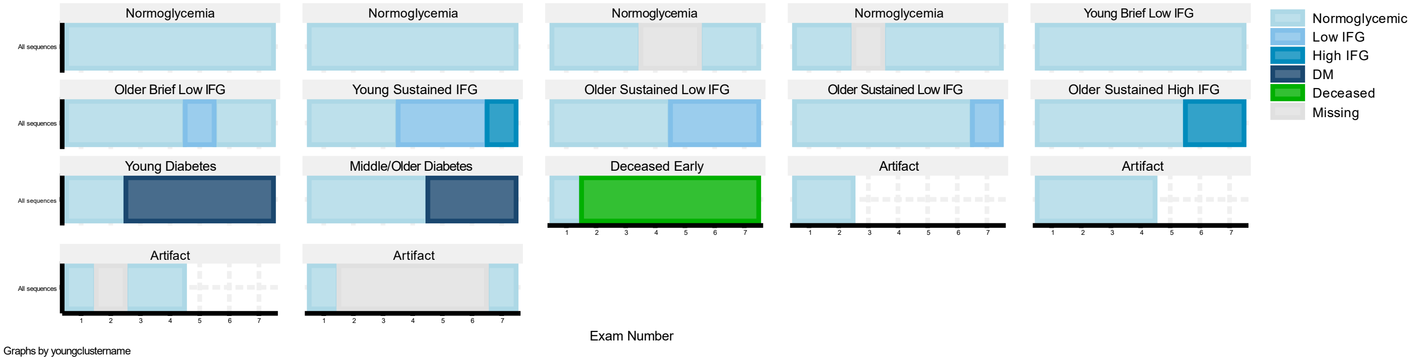

B. Chronogram

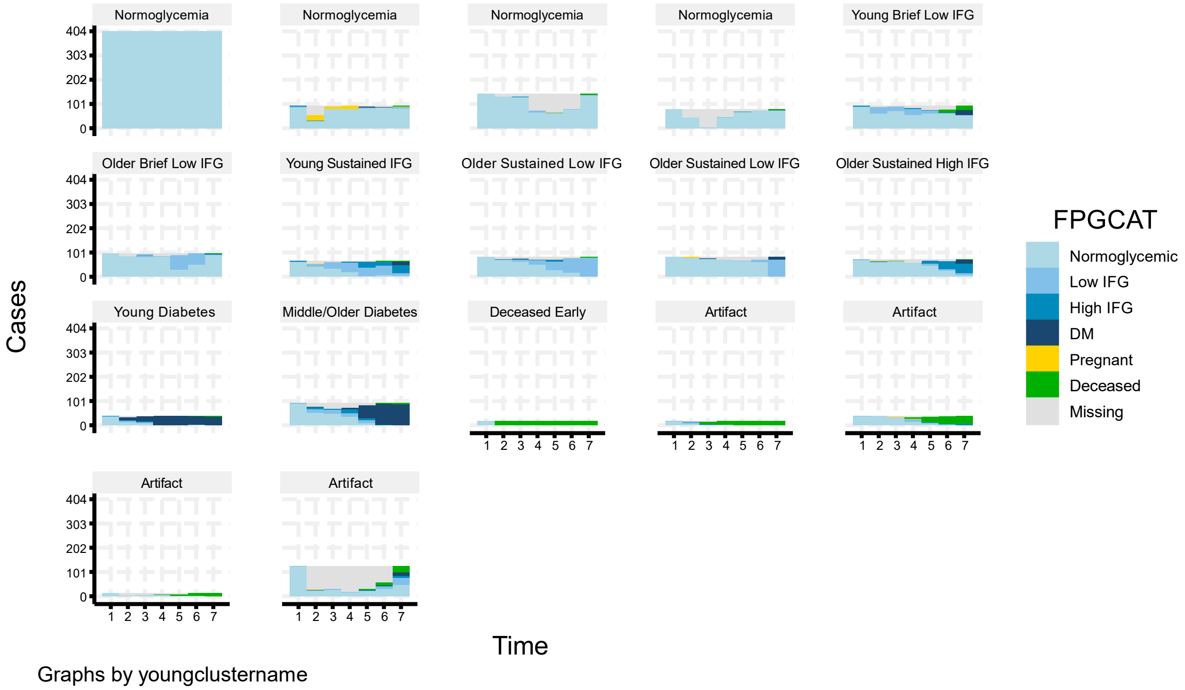

Results of sensitivity analysis with the younger group only and no age calibration. This analysis produced 17 total clusters as its optimal solution, with 8 main patterns of trajectory matching those seen in the main analysis (combining Middle and Older diabetes into a single cluster) as demonstrated in **A.** Modal plot and **B.** Chronoplot. In total, 69% (n=1420) of individuals landed in the same trajectory pattern as in the main analysis, with the remaining 31% (n=627) changing trajectory patterns: 2% (n=32) moved into artifact, 11% (n=215) moved out of artifact into a glycemic pattern, 9% (n=176) moved among IFG clusters, <1% (n=2) moved among diabetes clusters, 1% (n=29)

moved among artifact clusters, and the remaining 8% (n=173) moved between pattern types (either normoglycemic to IFG, IFG to normoglycemic, or Diabetes to normoglycemia/IFG).

© 2025 Arons AR et al. *JAMA Network Open.*

eFigure 7. Sensitivity analysis with theory based substitution penalty strategy

A. Matrix of theory based substitution penalties

	Normo-glycemia	Low IFG	High IFG	Diabetes	Pregnant	Deceased	Missing
**Normoglycemia**	0	1	2	3	1	1	1
**Low IFG**	1	0	1	1	1	1	1
**High IFG**	2	1	0	2	1	1	1
**Diabetes**	3	2	1	0	1	1	1
**Pregnant**	1	1	1	1	0	1	1
**Deceased**	1	1	1	1	1	0	
**Missing**	1	1	1	1	1	1	0

B. Modal Plot

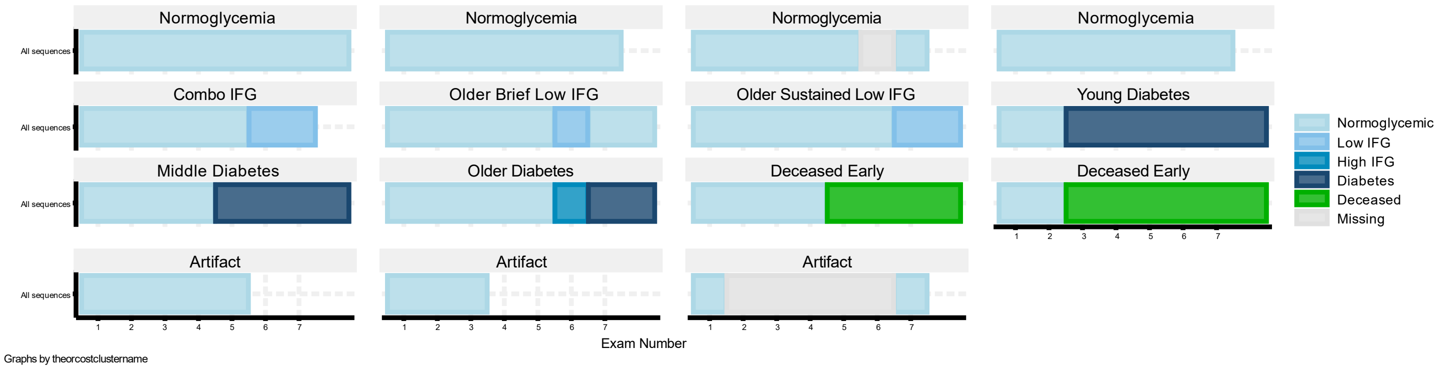

C. Chrono Plot

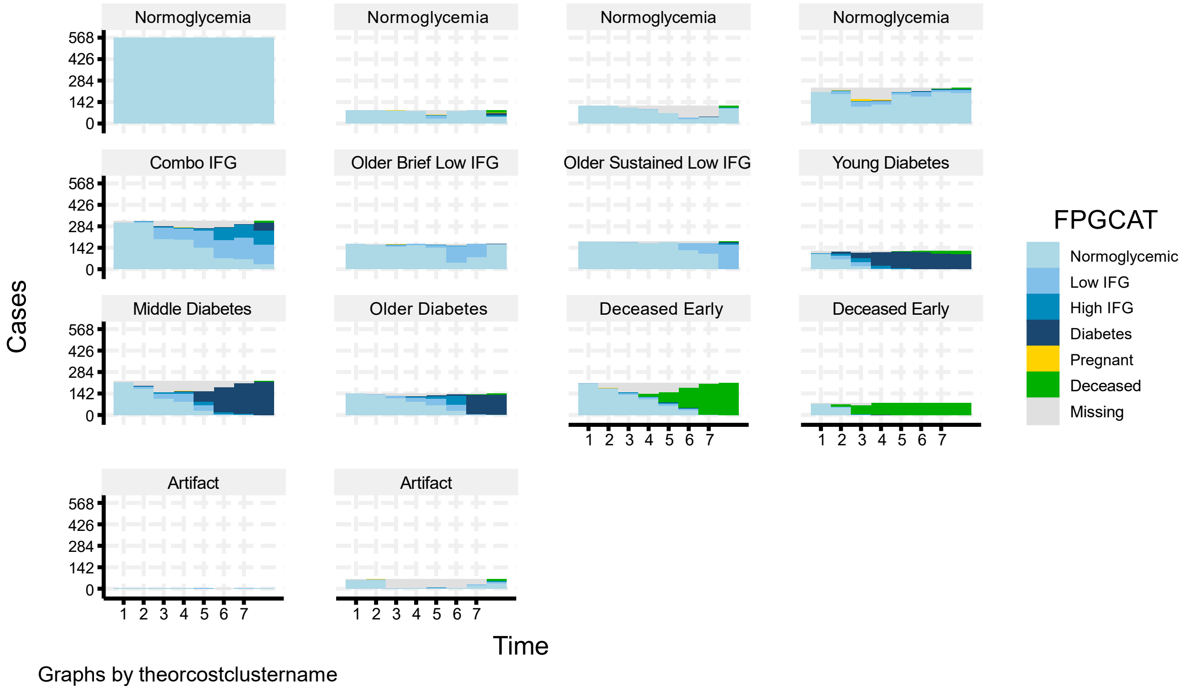

© 2025 Arons AR et al. *JAMA Network Open.*

Results of sensitivity analysis using an alternative theory-based substitution penalty strategy in which we fixed penalties for substitutions between states based on how different the states are from each other clinically. In the main analysis we used a data-driven method (transitions-based substitution matrix) which assigns penalties based on an algorithm where more frequent transitions such as normoglycemia to low IFG had lower cost than less frequent transitions such as normoglycemia to diabetes. **A.** Theory-based substitution matrix showing penalties assigned for substitutions in this analysis (e.g. if the penalty for substituting a normoglycemia value for a High IFG value would be 2, whereas Low IFG would be 1).

This sensitivity analysis resulted in 15 clusters instead of the original 18 cluster solution, with the chief difference being that multiple IFG clusters were collapsed into a single IFG cluster (e.g. young brief low, young sustained low, and older sustained high IFG), effectively losing some of the differentiation by IFG duration and timing. The other clusters represented the same patterns as in the main analysis. **B.** Modal plot and **C.** Chronoplot. 73.2% of individuals were grouped into the same cluster as in the main analysis (including those who went into the combined IFG cluster), indicating robustness of the original solution.

© 2025 Arons AR et al. *JAMA Network Open.*

eFigure 8. Sensitivity analysis with expanded definition of diabetes

A. Modal Plot

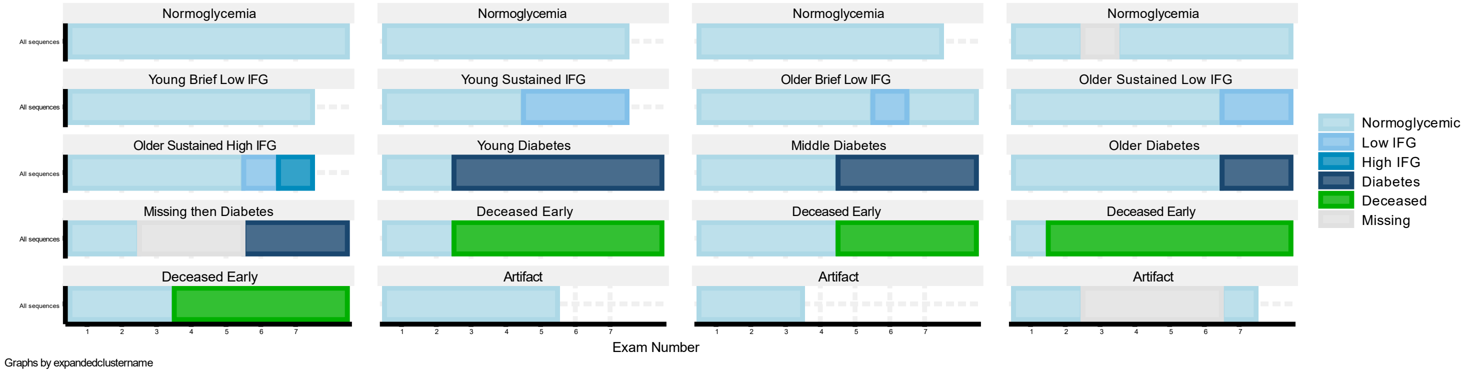

B. Chrono Plot

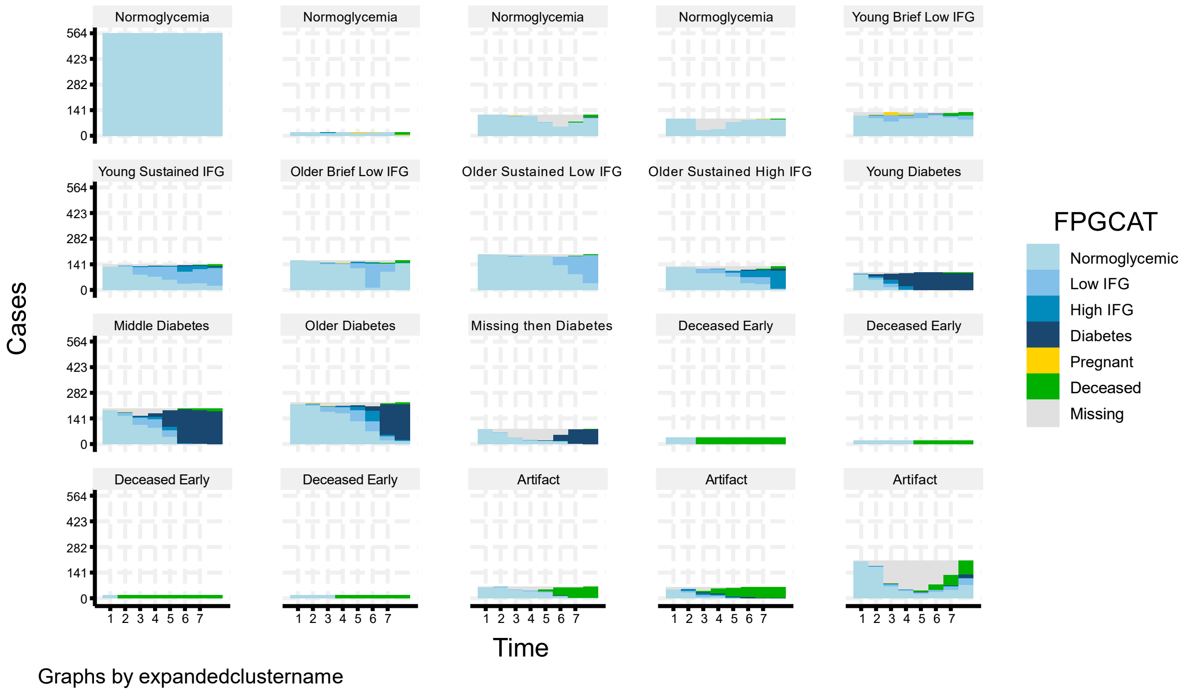

Results of sensitivity analysis with an expanded definition of diabetes, in which diabetes was considered as meeting criteria by *one* of: Oral Glucose Tolerance Test (exam years 10, 20, 25, 30); HgbA1c (exam years 20, 25, 30); or the main analysis criteria as FPG or antidiabetic medications. By year 35 there were 79 additional cases of diabetes detected by the broader criteria (in addition to 756 detected via the strategy of FPG and medication). The analysis produced 20 clusters as its optimal solution, all main patterns of trajectory matching those seen in the main analysis demonstrated in **A.** Modal plot and **B.** Chronoplot.

© 2025 Arons AR et al. *JAMA Network Open.*

In total, 3904 (83.4%) individuals landed in the same trajectory pattern as in the main analysis, with the remaining 780 (16.7%) changing trajectory patterns. Of the full sample population, 174 (3.7%) moved into unclassifiable clusters from meaningful patterns, 104 (2.2%) moved out of unclassifiable into a glycemic pattern, 230 (4.9%) moved among IFG clusters, 51 (1.1%) moved among diabetes clusters, 34 (0.7%) moved among unclassifiable clusters, and the remaining 187 (4.0%) moved between pattern types (either normoglycemic to IFG, IFG to normoglycemic, or diabetes to normoglycemia or IFG). 120 individuals (2.6%) moved newly into a diabetes cluster.

© 2025 Arons AR et al. *JAMA Network Open.*

eTable 1. Baseline characteristics of "Unclassifiable" Clusters

Cluster name	Full Sample	Missing then Diabetes	Normo. then deceased early	Artifact due to Missingness
**n**	4683	81	69	1,114
**% full sample in cluster**	100%	1.7%	1.4%	23.7%
**Sex (F)**	54%	57%	28%	49%
**Race/Ethnicity**				
Black	50%	77%	62%	60%
White	50%	23%	38%	40%
**Father's Education**				
Less than HS	17%	22%	13%	17%
HS	29%	28%	38%	31%
College or more	36%	27%	26%	28%
**Mother's Education**				
Less than HS	14%	14%	9%	15%
HS	40%	44%	45%	43%
College or more	38%	32%	39%	33%
**Family history of DM**				
Parent with hx DM	14%	27%	22%	14%
Sibling with hx DM	3%	4%	4%	3%
Any family hx DM	15%	30%	25%	15%
**Baseline BMI**	24.5 (5.0)	27.1 (5.1)	24.6 (5.5)	24.3 (5.0)

These clusters are more socially vulnerable than the overall sample.

© 2025 Arons AR et al. *JAMA Network Open.*

eTable 2. Full results of bivariate analysis with IFG categories defined as first IFG before or after age 35 (modeling potential screening for IFG at ages younger than 35)

	Young diabetes (40 or younger) n=144	Middle age diabetes (ages 40-50) n=232	Older diabetes (age 50 or older) n=380	No diabetes during study period n=3928
No IFG*	79 (55%)	83 (36%)	75 (20%)	2207 (56%)
IFG before age 35	57 (40%)	48 (21%)	56 (15%)	300 (8%)
First IFG at 35 and up	8 (6%)	101 (44%)	249 (66%)	1421 (36%)
	100%	100%	100%	100%

chi-squared p<0.001

*For people with diabetes, they were included as No IFG if they had no IFG exams prior to their first diabetes exam, even if they later had an IFG exam occurring after diabetes (as IFG screening would not have pre-identified them as at risk for diabetes). For those without diabetes, this category indicates no IFG during the entire study period

© 2025 Arons AR et al. *JAMA Network Open.*
