# Supplementary material for: Thirty-Year Glycemic Trajectories From Young Adulthood Through Middle Age
Source: JAMA Netw Open. 2025 Jun 26;8(6):e2517455. doi: 10.1001/jamanetworkopen.2025.17455 (PMC12203282; doi:10.1001/jamanetworkopen.2025.17455)
Supplement: Supplement 2. — Data Sharing Statement [file jamanetwopen-e2517455-s002.pdf]

Data Sharing Statement

Arons. Thirty-Year Glycemic Trajectories From Young Adulthood Through Middle Age. *JAMA Netw Open*. Published June 26, 2025. doi:10.1001/jamanetworkopen.2025.17455

Data
Data available: Yes
Data types: Deidentified participant data
How to access data: CARDIA data is available by request through the CARDIA coordinating center.
When available: With publication

Supporting Documents
Document types: None

Additional Information
Who can access the data: CARDIA data is available by request through the CARDIA coordinating center.
Types of analyses: For approved analyses
Mechanisms of data availability: Upon agreement with the CARDIA coordinating center
